# Ethnical discrimination in Europe: Field evidence from the finance industry

**DOI:** 10.1371/journal.pone.0191959

**Published:** 2018-01-29

**Authors:** Matthias Stefan, Felix Holzmeister, Alexander Müllauer, Michael Kirchler

**Affiliations:** 1 Department of Banking and Finance, Leopold-Franzens Universität, Universitätsstraße 15, A-6020 Innsbruck, Austria; 2 Centre for Finance, Department of Economics, University of Göteborg, SE-40530 Göteborg, Sweden; Mälardalen University, SWEDEN

## Abstract

The integration of ethnical minorities has been a hotly discussed topic in the political, societal, and economic debate. Persistent discrimination of ethnical minorities can hinder successful integration. Given that unequal access to investment and financing opportunities can cause social and economic disparities due to inferior economic prospects, we conducted a field experiment on ethnical discrimination in the finance sector with 1,218 banks in seven European countries. We contacted banks via e-mail, either with domestic or Arabic sounding names, asking for contact details only. We find pronounced discrimination in terms of a substantially lower response rate to e-mails from Arabic senders. Remarkably, the observed discrimination effect is robust for loan- and investment-related requests, across rural and urban locations of banks, and across countries.

## Introduction

The European Union (EU) is one of the most important destinations for immigrants from non-European and European countries, with 3.8 million people having immigrated to one of the member states in 2014 [1]. Lately, reports about the large number of asylum seekers fleeing their homes—1.2 million asylum applications in 2015 [2]—have been dominating Europe’s media landscape. At the same time Europe’s population is divided over its stance towards immigration from predominantly Muslim countries [3, 4]. However, not only Europe is facing a challenge from increasing immigration and therefore the question how to integrate immigrants from different countries and cultures has taken center stage in the political, societal, and economic debate in a globalized world. Successful economic integration of immigrants is a precondition for countries to benefit from their potential and a tool for avoiding conflicts. However, one of the main obstacles for integration is ethnical and racial discrimination. Each year, in Europe millions of people are facing discrimination and millions more are living under the threat of being discriminated against [5].

According to prior literature, there are two types of discrimination: First, there is the so called taste-based discrimination, i.e. discrimination due to mere personal prejudices [6]. Such discrimination due to ethnical prejudices should disappear in competitive environments and markets with low entry barriers where the exclusion of potential customers is costly to the agent [7]. Second, ethnical discrimination can also be rooted in statistical discrimination, where prejudices serve as a proxy for unobserved statistical differences [8, 9]. Accordingly, prejudices work as a kind of screening device based on the assumption that an agent’s ethnicity is correlated with socioeconomically relevant characteristics. In related research ethnical discrimination has been reported in different areas such as labor markets [10–14], education [15], housing markets [16], product markets [17, 18], public services [19–21], and loan sectors of the financial industry [22–25]. Thus, ethnical discrimination has been shown to be a persistent phenomenon in economic interactions [26]. However, despite the so called immigration crisis in Europe, we are only aware of few economic studies on ethnical discrimination in Europe [25, 27–29]. This is surprising given the prominence of the public debate as well as the relevance of immigration in Europe in general. For researchers and policy makers alike it is essential to acquire an encompassing understanding of ethnical discrimination and to measure the extent and occurrence of discrimination. Only this way it is possible to acquire informed policies that build on objective and reliable data [5].

In this paper we address the question whether discrimination of ethnical minorities exists in the European finance sector. Our study is motivated by (i) the relevance of migration in (European) politics and (ii) the central role the finance industry plays for the functioning of the economy and for satisfying the financial preferences of individuals. Unequal access to the finance sector due to discrimination might have severe economic consequences for the individual by potentially increasing disparities, wasting human resources, and causing poverty and social exclusion [7]. We contribute to the literature with the first cross-country field study of ethnical discrimination in the loan and investment sector in seven European countries. To get a more comprehensive picture we gather data on discrimination on either of the two pivotal domains of finance: the investment and the loan sector. First, different profits, especially given the zero interest environment, and different risks prevail in the two domains, such as counterparty risk in the loan sector and risk of illegal money or money laundering in the investment sector. For these reasons, banks’ employees might act differently in the two domains, depending on how the risk-return perception influences the willingness to serve customers. Second, for the individual customer, discrimination in both domains can have severe effects on future wealth (e.g., pension savings) and access to loans (e.g., for housing or to start a new business). For these reasons, it is important to have a comprehensive view on discrimination in the finance industry.

## Experimental design

This study was approved by the Ethics Committee of the University of Innsbruck. We conducted a field experiment [30] with the method of e-mail correspondence as used by [31] and [21], among others. In particular, we gathered 1,218 observations from a randomly selected sample of banks in seven countries in Europe. The numbers of e-mail queries distributed within each country are provided in Table A in S1 Appendix. The treatment variable is the ethnicity of the sender (ethnicity). We sent half of the e-mails with a domestic sounding sender’s name while the other half has been sent with a foreign sounding sender’s name, most likely to be associated with the Middle East. The name appears in the e-mail address as well as in the signature of the e-mail to make it as salient as possible. A foreign sounding name is not a clear indicator whether the sender is a non-native or a native with ethnical background. However, in previous studies on ethnical discrimination on labor markets, no differences was found in discrimination between natives with ethnical background and non-natives as well as between first- and second-generation migrants [32, 33]. All e-mails have been sent out in the local languages of the respective countries. We included a doctor’s degree for all names in the e-mails, intended to serve as an indicator for high socioeconomic status. The e-mail is depicted in Fig A in S1 Appendix. In addition, we varied the type of request (type) which is either loan-related or investment-related. Again, the type of request was mentioned in the short body as well as the subject field to make it as salient as possible. Importantly, the message only asked for contact details, not for any specific information about investment or financing opportunities, and, thus, could be answered by any employee. The additional information, which is necessary for distinguishing between the two types of inquiry, was phrased as vague as possible. For this reason, it should neither allow to extract more information than the respective type of inquiry, nor influence the fact that e-mails could be answered directly by any employee. Except for the name and the type of request, the e-mails were identical. Further details on the methods applied are outlined in Section A of S1 Appendix and potential ethical issues of the study are discussed in Section B of S1 Appendix.

The primary variable of interest is the response rate to our e-mail request. Only personal replies are considered as responses; automatic replies are not. We do not analyze the content of the responses. The reason is that the e-mail inquiries only ask for contact details, so that there is not much room for variety in the replies. We further discuss the interpretation of e-mail responses as a proxy for discrimination behavior in the Discussion section.

## Results

**Fig 1:**
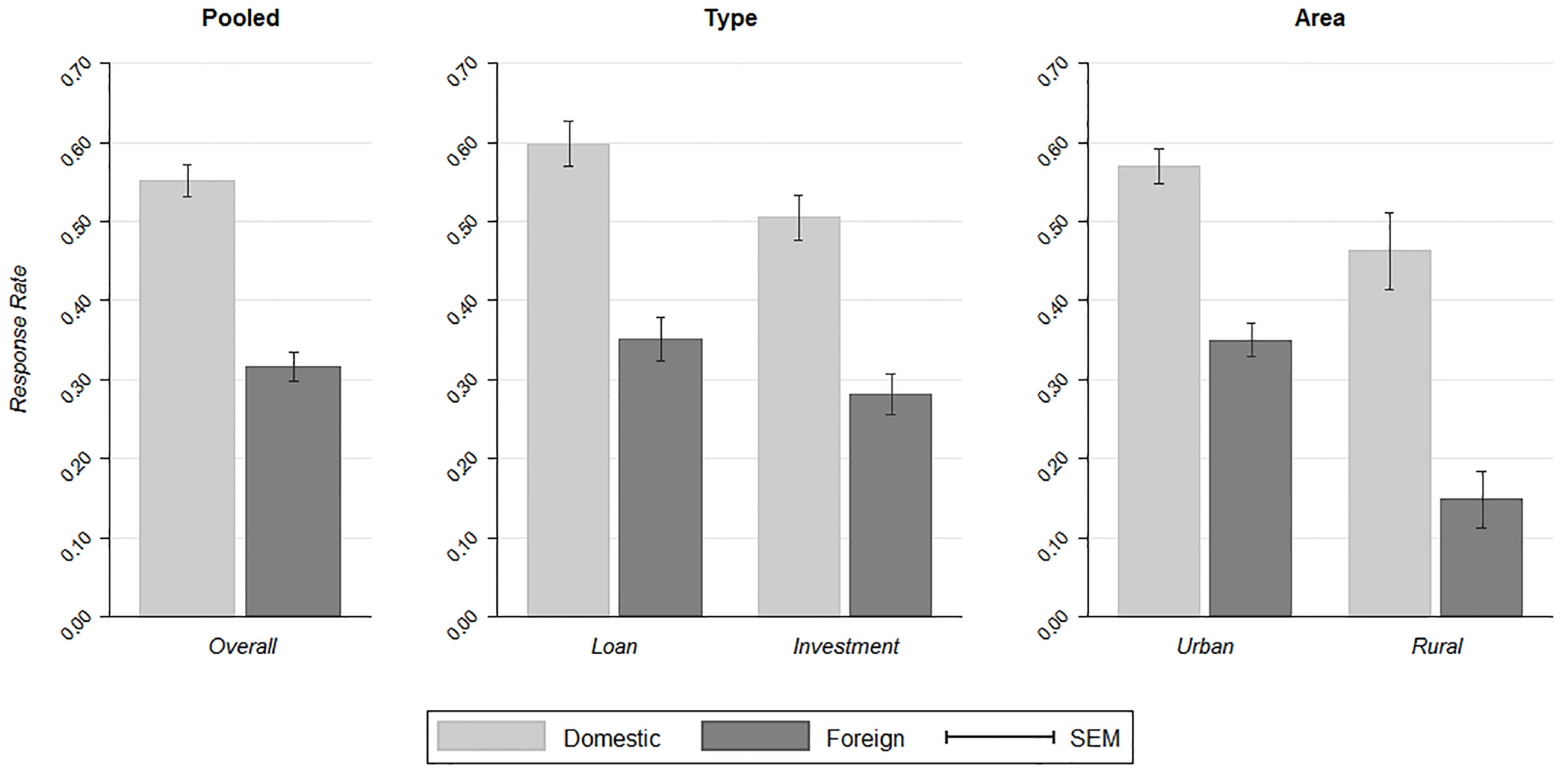
Response rates to e-mail requests in the pooled data (left panel), separated by type (middle panel), and separated by area (right panel).

Overall, we received 529 replies to the 1,218 e-mail inquiries, which amounts to a response rate of 43.4% (sem = 1.4%) in the pooled data. E-mails with domestic sounding names triggered a response rate of 55.2% (sem = 2.0%), whereas e-mails with Arabic sounding names were replied in only 31.6% (sem = 1.9%) of the cases. The response rates are presented in the left panel of Fig 1. The difference between response rates to e-mails with domestic sounding and Arabic sounding names is statistically significant (Pearson’s *χ*^2^-test; *n* = 1,218, *χ*^2^(1) = 68.587, *p* < 0.001). Arguably, the difference of 23.5 percentage points (*pp*) in response rates is of economically significant magnitude, too.

The observed difference in responses to e-mail queries, which we consider being a strong indication of ethnical discrimination, is robust to the type of queries. Discrimination rates of investment- and loan-related requests are depicted in the middle panel of Fig 1 and in the left panel of Table B in S1 Appendix. The pattern of lower response rates to e-mails with Arabic sounding names is persistent in either type of query. With 59.8% (sem = 2.8%) and 50.5% (sem = 2.9%) the response rates for domestic sounding names are substantially higher than for Arabic sounding names with 35.1% (sem = 2.7%) and 28.1% (sem = 2.6%), respectively. The differences of 24.7*pp* and 22.3*pp* are statistically significant (Pearson’s *χ*^2^-tests; loan-related request: *n* = 611, *χ*^2^(1) = 37.439, *p* < 0.001; investment-related request: *n* = 607, *χ*^2^(1) = 31.743, *p* < 0.001). Notably, the difference in difference, i.e. the difference in the discrimination rates, between both types of requests is not statistically significant (Permutation test on differences in response rates, *p* = 0.689). Thus, there is no difference in the discrimination of requests with Arabic sounding names between the two sectors of the finance industry. We consider this being the first strong indicator for the robustness of our findings.

The observed discrimination effect is also robust irrespective of whether the banks are situated in urban or rural areas (area). It is commonly well known, that overall political attitudes differ systematically between urban and rural areas: on average, urban citizens tend to be more politically oriented to the left compared to rural citizens [34]. More importantly, it has been found that rural citizens tend to have more negative views on immigration and to favor restrictions of immigration [35–37]. For these reasons, we further investigate discrimination in these two areas. In what follows, rural areas are defined as places with less than 10,000 citizens. Although it is difficult to objectively classify regions as urban or rural, it is reassuring that the reported effect is robust qualitatively as well as quantitatively for a reasonable range of specifications of the variable area (details are provided in Table C in S1 Appendix). The response rates for banks in urban and rural areas are depicted in the right panel of Fig 1 and in the right panel of Table B in S1 Appendix. The response rates to e-mails with domestic sounding names are 57.0% (sem = 2.2%) and 46.2% (sem = 4.9%) in urban and rural areas, respectively. In contrast, e-mails with Arabic sounding names show substantially lower response rates of 35.0% (sem = 2.1%) and 14.9% (sem = 3.6%), respectively. Thus, the discrimination effect is observable in both areas, with a 22.0*pp* and 31.4*pp* lower response rates to e-mails from Arabic sounding senders. These differences in response rates are highly significant (urban areas: *χ*^2^(1) = 49.470, *p* < 0.001; rural areas: *χ*^2^(1) = 23.837, *p* < 0.001). We observe a higher level of response rates for banks in urban areas, but, again, the difference in differences, i.e. the difference in discrimination rates, between rural and urban areas is not statistically significant (permutation test on differences in response rates, *p* = 0.217). Therefore, the observed discrimination effect is robust across banks’ locations. This is a striking result and serves as another strong indicator for the robustness of our findings. Notably, our findings are also consistent if the discrimination effect is separated by types of requests as well as location of banks, as shown in Fig B in S1 Appendix.

Finally, Fig C in S1 Appendix highlights the response rates for each of the countries in the random sample. The discrimination effect is robust and persistent across all country sub-samples with substantial differences in response rates between domestic and Arabic sounding names, ranging from 10.1% (the Netherlands) to 83.7% (Finland). The differences in response rates are statistically significant at the 1%-level in all countries except for Denmark and the Netherlands. Response rates and differences and the corresponding *χ*^2^ test statistics are provided in Table D in S1 Appendix for each country separately. We consider the consistency of our findings in the country sub-samples as a third indicator of their robustness.

Logit regressions of the response on ethnicity, area, type and their interaction effects, controlled for country effects, are reported in Table E in S1 Appendix and corroborate the consistency of all results.

## Discussion

We conclude that the discrimination effect is robust across the type of request, the location of banks, and the different countries. These findings indicate that the reported discrimination effect is universal across different domains. First, the risk profiles and profit margins banks face might considerably differ between investment- and financing related customer relations. For instance, while banks typically face counterparty risks when issuing loans, existing regulation pose legal risks when investing potentially dirty money or illegal income. Second, within a country urban and rural areas are expected to differ in cultural and societal aspects as well. In general, urban citizens vote more for left-wing parties and have a more positive view on migration and vice versa for rural citizens. Therefore, more pronounced discriminating behavior might be expected to be found in rural areas with more people sharing negative views towards immigrants. Finally, countries differ in their political and societal culture, including difference such as in financial regulations, discrimination legislation, social discussion, political sentiment, etc. All those differences might influence discrimination behavior on the individual level in different ways.

Given the potentially considerable impact of different business domains as well as differences in the cultural, societal, and political environment, it is remarkable that neither of these differences change the observed discrimination effect in the finance industry found in our study. We consider this robustness as a substantial corroboration of our conclusion that the European finance sector is facing marked discrimination against customers with Arabic ethnicity.

It is noteworthy to bear in mind a basic trade-off in the experimental design choice of our study: Given the early stage of contacting a bank by asking for contact details only and the inclusion of a Dr.’s degree as signal of high socioeconomic status, we provide a rather conservative measure of ethnical discrimination. Furthermore, our experimental design aims at high external validity by observing bank employees’ behavior in a natural setting handling a common inquiry. However, the conservative approach and external validity come at the cost of not being able to explain the detailed mechanisms that drive our findings. That is, we cannot reasonably distinguish between statistical and taste-based discrimination given our e-mail queries. It is possible that taste-based prejudices play a role in the observed discrimination behavior. We cannot conclusively rule this out or confirm it given that the subjects’ individual preferences with regards to immigrants remain unknown in our study. Likewise, statistical discrimination might play a role in the observed discrimination behavior on the basis of potential higher expected legal and opportunity costs in case of foreign customers, such as if different regulatory frameworks are expected to apply or if tax avoidance issues might be apprehended. Thus, both, statistical and taste-based discrimination, can be potential explanations of the discrimination effects. However, it is noteworthy that institutions are contacted at a very early level of the bank-customer relationship and that there is no information in the e-mail apart from the investment- or loan-relatedness of the request. Therefore, bank employees do not have much information about the potential customer to build statistical discrimination on. For instance, it is not known whether the person is a citizen of the respective country, which would neglect higher costs due to different legal frameworks or higher risks of illegal money or tax avoidance and, therefore, would minimize justification of statistical discrimination at least in the finance domain. However, even though it stands to reason to assume that taste-based discrimination plays an important role in the discrimination of potential bank customers, we still cannot conclusively determine the type of discrimination prevalent in our study. This is an open question valuable for future research.

We deem our result important for two reasons: First, the observed discrimination effect can have potentially but severe economic consequences. Unequal access to the finance sector implies potential social and economic exclusion of minorities, inferior economic opportunities and, in turn, might lead to failing integration and aggravate economic disparities. Second, our findings are relevant for policy makers, since prevailing competition and low levels of entry barriers in the banking sector do not suffice to counter discrimination and to increase economic integration of immigrants. The banking sector is already highly competitive and highly regulated, but our study shows that immigrants have difficulties approaching banks even before potential legal and economic barriers become relevant. European countries heavily regulate the financial sector to ensure standardized high-quality service of customers, because of the seriousness of potential negative effects, which painfully came to mind again after the financial crisis. However, if existing discrimination cuts off potential foreign customers before such regulations become effective, this should be considered a serious issue for the affected individuals and societies as a whole.

## Supporting information

S1 Appendix Supplementary information. (PDF)
