# Supplementary material for: Ethnical discrimination in Europe: Field evidence from the finance industry
Source: PLoS One. 2018 Jan 29;13(1):e0191959. doi: 10.1371/journal.pone.0191959 (PMC5788365; doi:10.1371/journal.pone.0191959)
Supplement: S1 Appendix — (PDF) [file pone.0191959.s001.pdf]

# Ethnical Discrimination in Europe: Field Evidence from the Finance Industry

Matthias Stefan[1], Felix Holzmeister[1], Alexander Müllauer[1], and Michael Kirchler[*,1,2]

[1] Department of Banking and Finance, Leopold-Franzens Universität, Universitätsstraße 15, A-6020 Innsbruck, Austria

[2] Centre for Finance, Department of Economics, University of Göteborg, SE-40530 Göteborg, Sweden

[*] Corresponding Author (michael.kirchler@uibk.ac.at)

## Supporting Information

### A. Materials and Methods

In our study, we contacted a randomly selected sample of 1,229 banks in Austria, Germany, Belgium, the Netherlands, Denmark, Sweden, and Finland. We received automatic mail error replies for 11 e-mails. These observations were discarded, leaving us with a sample of 1,218 data points. The number of e-mail queries are provided in Table A.

To make the name of the sender as salient as possible, the sender's name appeared both in the body of the address field as well as in the e-mail query. The domestic sounding names for each of the countries were picked as a random combination from the respective 50 most common first and last names. We checked whether there are name hits in search engines and social media platforms for the particular name combinations that were chosen. To further reduce the risk of possible name hits we included an abbreviated middle initial. In particular, domestic sounding names used in either of the seven countries in the random sample were Lukas E. Gruber (Austria), Niels P. Goossens (Belgium), Oscar A. Nielsen (Denmark), Antti W. Mäkelä (Finland), Peter N. Müller (Germany), Arjen B. de Jong (Netherlands), and Per O. Norberg (Sweden). The foreign names were either Muhammad B. Atallah or Muhammad J. Atallah, i.e. typical Arabic sounding names. Two different abbreviated middle initials have been used for the Arabic sounding name, because of the e-mail provider's restriction regarding the number of e-mails sent per day. The e-mail is schematically depicted in Fig A.

| | |
|---|---|
| From: | [domestic name/foreign name] |
| | [firstname.middle-initial.lastname@gmail.com] |
| Subject: | Contact details regarding [loan/investment] |

Dear Sir or Madam,

because of occupational and financial changes, I am thinking about a possible diversification in my private [loan/investment] portfolio. Therefore, I kindly ask you about contact details to further discuss this issue. Thank you very much for your help.

Kind regards,

Dr. [domestic name/foreign name]

**Fig A.** E-mail message sent to banks.

The e-mail template has been written in German with the help of Austrian bankers. In the next step, for each country the e-mail has been translated into the official national language with the help of two independent native speakers. If a country has more than one official language, individual institutions were approached in the language that is spoken primarily in the respective region. Institutions were contacted via their official information addresses. To ensure that e-mails are not forwarded to a headquarter, we only approached institutions and subsidiaries with a unique contact e-mail address. All e-mails have been sent out at the same day. The time frame of the data collection was five days (a working week), ranging from May 9, 2016 to May 13, 2016. We sent a second e-mail to those banks that replied to the query, with a note that the issue has been resolved and that their service is very much appreciated but no longer needed.

**Table A.** Number of observations in each country.

| | AUT | BEL | DAN | FIN | GER | NED | SWE | Total |
|---|---|---|---|---|---|---|---|---|
| *Domestic/Loan* | 37 | 37 | 25 | 16 | 96 | 48 | 47 | 306 |
| *Domestic/Investment* | 31 | 37 | 26 | 14 | 97 | 48 | 52 | 305 |
| *Foreign/Loan* | 32 | 38 | 25 | 14 | 99 | 48 | 49 | 305 |
| *Foreign/Investment* | 28 | 38 | 27 | 15 | 101 | 45 | 48 | 302 |
| Total | 128 | 150 | 103 | 59 | 393 | 189 | 196 | 1,218 |
| in % | 10.5 | 12.3 | 8.5 | 4.8 | 32.3 | 15.5 | 16.1 | 100.0 |

*Notes:* Abbreviations for countries are used as follows: AUT for Austria, BEL for Belgium, DAN for Denmark, FIN for Finland, GER for Germany, NED for the Netherlands and SWE for Sweden.

## B. Ethics

This study was approved by the Ethics Committee of the University of Innsbruck. Furthermore, we took all measures to ensure full confidentiality. However, there are two arguments that might be raised against the ethical justification of the study, which we want to discuss in what follows. For further details on ethical issues in field studies on discrimination see [1].

The first argument is that banks were contacted and, thereby, their time was consumed in answering our e-mails. Even though we fully agree that it is important to cautiously consider possible costs and effects that field studies incur on the subjects under investigation, we still consider our study to be ethically valid for the following reasons: First and foremost, it is our opinion that the possible costs for the banks are outweighed by the benefit of the study for the society as a whole. It is important for societies to acquire a accurate view on discrimination based on objective data to acquire informed policies, since the costs stemming from failed integration and discrimination can be severe for individuals and societies as a whole. Second, we consider our e-mail inquiry to incur only little cost and effort to the respective banks' employees. The e-mail inquiry can be answered straightforwardly without requiring to gather further information. To reduce additional effort by checking back whether senders need more information or further help, we sent out the second e-mail described in Section A that the issue has been resolved and no further information is necessary.

The second argument is that banks were contacted with fake names and inquiries that are specifically designed for this study. As experimental economists we are very concerned with deception of subjects. However, field studies on discrimination are impossible to conduct without a minimal form of misinformation. Moreover, utilizing deceptive elements in our experimental design does not force subjects into situations they are not facing in their everyday environment. Rather, this kind of information is used to observe common behavior in the workplace. Again, we argue that the costs incurred with this practice are justified by the high information content of our study for society and policy makers.

## C. Supplementary Analyses

Table B summarizes the response rates to e-mails for domestic and Arabic sounding names, separated for the type of request (loan- or investment-related; left panel) as well as for the rurality of the respective bank's location (urban or rural; right panel) as discussed in the main text. The results with regards to AREA refer to a threshold of 10,000 inhabitants (different thresholds are discussed below). We find a pronounced and significant discrimination effect in terms of lower response rates to e-mails with Arabic sounding names, irrespective of the type of request and the banks' locations. Furthermore, we find no significant differences in the discrimination rates between the two types of requests and urban and rural areas, respectively.

**Table B.** Response rates for factorial combinations of ETHNICITY/TYPE and ETHNICITY/AREA.

| | TYPE | | | AREA | | |
|---|---|---|---|---|---|---|
| | *Loan* | *Investment* | *Difference* | *Urban* | *Rural* | *Difference* |
| *Domestic* | 0.598 | 0.505 | $-0.093^{*}$ | 0.570 | 0.462 | $-0.108^{*}$ |
| | (0.028) | (0.029) | | (0.022) | (0.049) | |
| *Foreign* | 0.351 | 0.281 | $-0.069$ | 0.350 | 0.149 | $-0.201^{**}$ |
| | (0.027) | (0.026) | | (0.021) | (0.036) | |
| *Difference* | $-0.247^{***}$ | $-0.223^{***}$ | $0.024^{\text{n.s.}}$ | $-0.220^{***}$ | $-0.314^{***}$ | $-0.093^{\text{n.s.}}$ |

*Notes:* Standard errors of the mean are reported in parenthesis. *, **, and *** denote the 5%, 1%, and 0.1% significance level, respectively; *n.s.* refers to 'not significant'. Significance indications are based on $\chi^2(1)$ statistics for column and row differences and permutation tests (on differences in frequencies; 10,000 permutations) for differences-in-differences, respectively.

Although it is not the primary focus of our paper, two additional results may be noteworthy: First, the difference in response rates between the loan- (47.5%, SEM = 2.0%) and investment-related requests (39.4%, SEM = 2.0%) of 8.1*pp* is statistically significant ($\chi^2(1) = 8.110$, $p = 0.004$). Furthermore, the difference in response rates between the loan- and investment-related request for domestic sounding names (9.3*pp*) turns out to be statistically significant ($\chi^2(1) = 5.355$, $p = 0.021$), although the difference for Arabic sounding names (6.9*pp*) does not significantly differ from zero ($\chi^2(1) = 3.376$, $p = 0.066$). The lower response rates in the investment domain might be driven by higher potential revenues from the loan business or by banks' current excess liquidity in the Euro area, both potentially rendering the investment business less attractive for banks. Second, we report level effects in response rates for the variable AREA. The response rates of 46.0% (SEM = 1.6%) in urban areas is significantly larger than the response rate of 30.9% (SEM = 3.2%) in rural areas ($\chi^2(1) = 15.896$, $p < 0.001$). The difference in response rates between urban and rural areas persists for both domestic ($d = -10.8pp$, $\chi^2(1) = 4.134$, $p = 0.042$) and foreign sounding names ($d = -20.1pp$, $\chi^2(1) = 15.774$, $p < 0.001$). This might be due to the fact that e-mail inquiries are more common in urban areas. However, both explanations, for the differences in response rates over types as well as areas, are only speculative and our data set does not allow to infer where these level effects stem from.

In addition, the observed discrimination effect also persists for the interaction of both variables, TYPE and AREA, as can be seen in Fig B. The response rate for loan-related requests in urban areas is 60.2% (SEM = 3.0%) for domestic sounding names, compared to 39.2% (SEM = 3.1%) for Arabic sounding names ($\chi^2(1) = 22.508$, $p < 0.001$). For investment-related requests in urban areas, response rates for domestic and Arabic sounding names are 53.7% (SEM = 3.2%) and 30.9% (SEM = 2.9%), respectively ($\chi^2(1) = 26.763$, $p < 0.001$). In rural areas, loan-related requests sent with a domestic sounding name trigger a response rate of 57.4% (SEM = 7.3%) as compared to a response rate of 16.4% (SEM = 5.0%) for Arabic sounding names ($\chi^2(1) = 18.730$, $p < 0.001$). For investment-related requests in rural areas, response rates for domestic and Arabic sounding names equal 37.3% (SEM = 6.3%) and 13.0% (SEM = 5.0%), respectively ($\chi^2(1) = 7.769$, $p = 0.005$). Thus, the observed discrimination effect reported in the main text is also robust to the interaction of the factorial combinations, which further corroborates our findings.

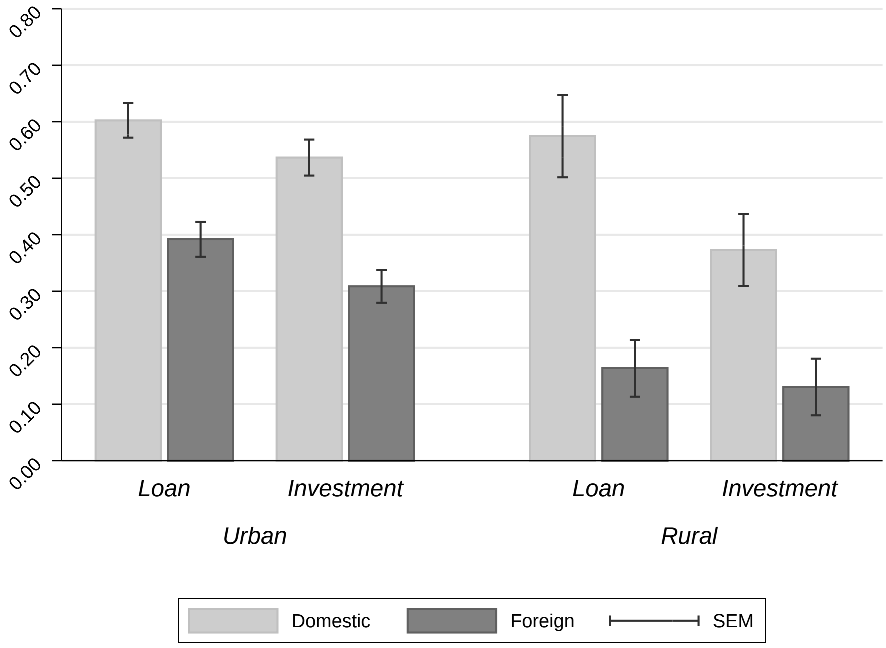


**Fig B.** Response rates for domestic and foreign sounding names separated for the type of request (loan/investment) as well as the rurality of the banks' location (urban/rural).

Results reported with respect to the rurality of the banks' location are based on a threshold of 10,000 inhabitants. Accordingly, a bank is considered being located in an urban area whenever the number of inhabitants of the location has at least 10,000 inhabitants; otherwise, the bank is considered being located in a rural area. Although a threshold of 10,000 inhabitants appears to be reasonable for differentiating between urban and rural areas in Europe, the classification has to remain somewhat arbitrary. For this reason, we test for the robustness of the reported results by varying the threshold between 5,000 and 25,000 inhabitants in steps of 2,500 and re-estimating all results reported for any

of these levels as reported in Table C. It is reassuring that the effect reported in the main text as well as above are robust for this range of thresholds, i.e. discrimination effects remain highly significant and at similar levels, while differences in discrimination rates remain insignificant.

**Table C.** Robustness checks for differences in response rates separated for the rurality of a banks' location for different thresholds for defining urban areas.

***Panel A: Area = urban***

| *Threshold* | *5,000* | *7,500* | *10,000* | *12,500* | *15,000* | *17,500* | *20,000* | *22,500* | *25,000* |
|---|---|---|---|---|---|---|---|---|---|
| *Domestic* | 0.565 | 0.564 | 0.570 | 0.575 | 0.584 | 0.579 | 0.577 | 0.573 | 0.574 |
| | (0.021) | (0.022) | (0.022) | (0.023) | (0.023) | (0.024) | (0.024) | (0.025) | (0.026) |
| *Foreign* | 0.341 | 0.347 | 0.350 | 0.355 | 0.363 | 0.360 | 0.360 | 0.363 | 0.370 |
| | (0.020) | (0.021) | (0.021) | (0.022) | (0.023) | (0.023) | (0.023) | (0.024) | (0.025) |
| *Abs. Diff.* | −0.224 | −0.217 | −0.220 | −0.220 | −0.221 | −0.219 | −0.217 | −0.209 | −0.205 |
| *Rel. Diff.* | −0.396 | −0.384 | −0.387 | −0.383 | −0.378 | −0.378 | −0.376 | −0.365 | −0.356 |
| $\chi^2(1)$ | 55.443 | 49.742 | 49.470 | 46.473 | 44.090 | 41.749 | 39.526 | 34.780 | 31.942 |
| $p$-value | $< 0.001$ | $< 0.001$ | $< 0.001$ | $< 0.001$ | $< 0.001$ | $< 0.001$ | $< 0.001$ | $< 0.001$ | $< 0.001$ |

***Panel B: Area = rural***

| *Threshold* | *5,000* | *7,500* | *10,000* | *12,500* | *15,000* | *17,500* | *20,000* | *22,500* | *25,000* |
|---|---|---|---|---|---|---|---|---|---|
| *Domestic* | 0.438 | 0.469 | 0.462 | 0.466 | 0.459 | 0.483 | 0.497 | 0.514 | 0.515 |
| | (0.063) | (0.056) | (0.049) | (0.044) | (0.040) | (0.038) | (0.036) | (0.034) | (0.033) |
| *Foreign* | 0.071 | 0.128 | 0.149 | 0.176 | 0.184 | 0.207 | 0.216 | 0.226 | 0.224 |
| | (0.035) | (0.036) | (0.036) | (0.033) | (0.031) | (0.031) | (0.030) | (0.029) | (0.028) |
| *Abs. Diff.* | −0.366 | −0.341 | −0.314 | −0.290 | −0.275 | −0.276 | −0.281 | −0.288 | −0.291 |
| *Rel. Diff.* | −0.837 | −0.727 | −0.679 | −0.623 | −0.600 | −0.571 | −0.565 | −0.560 | −0.565 |
| $\chi^2(1)$ | 20.467 | 23.427 | 23.837 | 25.303 | 27.351 | 29.305 | 32.565 | 37.688 | 41.322 |
| $p$-value | $< 0.001$ | $< 0.001$ | $< 0.001$ | $< 0.001$ | $< 0.001$ | $< 0.001$ | $< 0.001$ | $< 0.001$ | $< 0.001$ |

***Panel C: Difference in discrimination rates between urban and rural areas***

| *Threshold* | *5,000* | *7,500* | *10,000* | *12,500* | *15,000* | *17,500* | *20,000* | *22,500* | *25,000* |
|---|---|---|---|---|---|---|---|---|---|
| *Urban* | −0.224 | −0.217 | −0.220 | −0.220 | −0.221 | −0.219 | −0.217 | −0.209 | −0.205 |
| *Rural* | −0.366 | −0.341 | −0.314 | −0.290 | −0.275 | −0.276 | −0.281 | −0.288 | −0.291 |
| *Diff.* | −0.142 | −0.124 | −0.093 | −0.070 | −0.054 | −0.057 | −0.064 | −0.079 | −0.086 |
| $p$-value | 0.142 | 0.132 | 0.225 | 0.315 | 0.404 | 0.357 | 0.289 | 0.194 | 0.141 |

*Notes:* Standard errors of the mean are reported in parenthesis. Significance indications are based on $\chi^2(1)$ statistics for column and row differences and permutation tests (on differences in frequencies; 10,000 permutations) for differences-in-differences, respectively.

Fig C shows the response rates to the e-mail queries for domestic and Arabic sounding names, separated for the seven countries in our sample. Table D summarizes response rates for domestic and Arabic sounding names, absolute and relative differences, as well as $\chi^2(1)$ statistics and the corresponding $p$-values. The difference between domestic and Arabic sounding names is persistent in all countries even if the magnitude of effect sizes varies considerably.

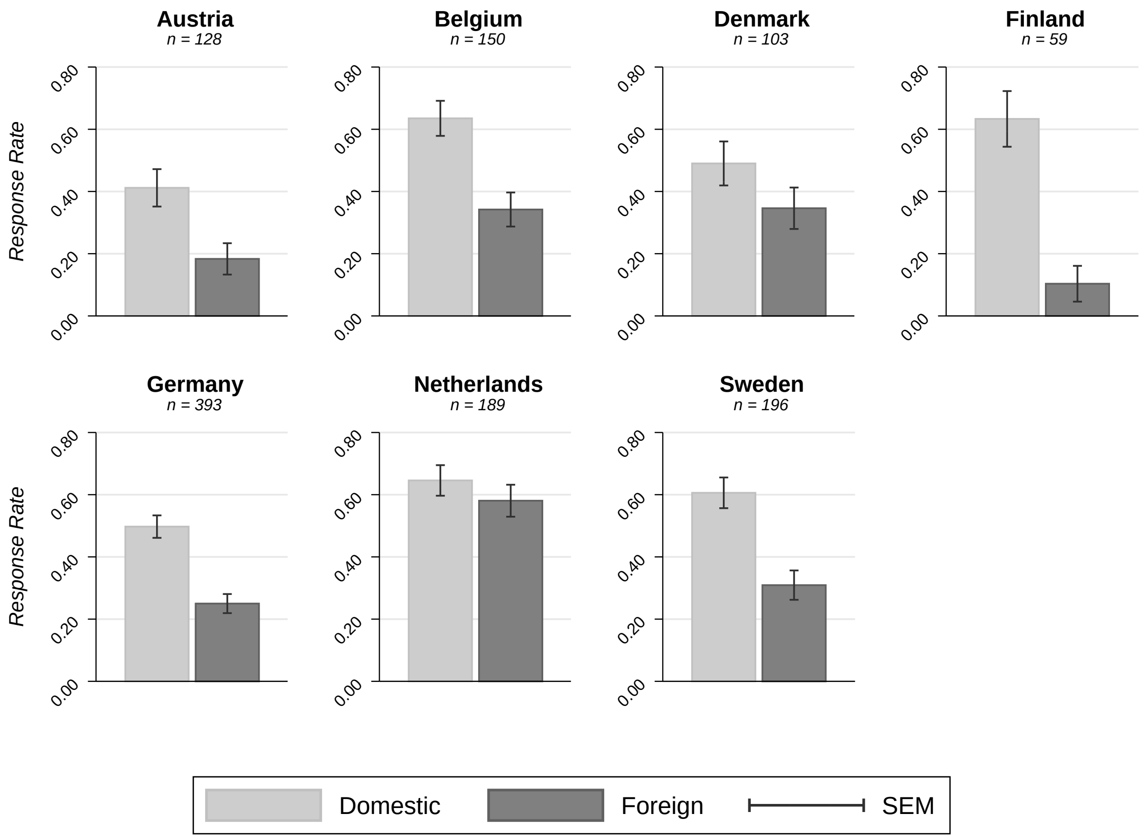


**Fig C.** Response rates for domestic and foreign sounding names separated for the seven countries of the random sample.

**Table D.** Response rates for domestic and foreign sounding names and differences for each of the seven countries in the sample. Test statistics for Pearson's $\chi^2$-tests and estimates of the statistical power for a two-sample proportions test based on a significance criterion of $\alpha = 5\%$ are reported.

| Country | AUT | BEL | DAN | FIN | GER | NED | SWE | Total |
|---|---|---|---|---|---|---|---|---|
| Domestic | 0.412 | 0.635 | 0.490 | 0.633 | 0.497 | 0.646 | 0.606 | 0.552 |
| Foreign | 0.183 | 0.342 | 0.346 | 0.103 | 0.250 | 0.581 | 0.309 | 0.316 |
| Abs. Difference | −0.229 | −0.293 | −0.144 | −0.530 | −0.247 | −0.065 | −0.297 | −0.235 |
| Rel. Difference | −0.556 | −0.461 | −0.294 | −0.837 | −0.497 | −0.101 | −0.490 | −0.427 |
| $\chi^2(1)$ | 7.851 | 12.887 | 2.197 | 17.706 | 25.749 | 0.847 | 17.378 | 68.587 |
| $p$-value | 0.005 | $< 0.001$ | 0.138 | $< 0.001$ | $< 0.001$ | 0.357 | $< 0.001$ | $< 0.001$ |
| Stat. Power | 0.490 | 0.733 | 0.181 | 0.871 | 0.957 | 0.098 | 0.842 | $> 0.999$ |
| $N$ | 128 | 150 | 103 | 59 | 393 | 189 | 196 | 1,218 |

*Notes:* Abbreviations for countries are used as follows: AUT for Austria, BEL for Belgium, DAN for Denmark, FIN for Finland, GER for Germany, NED for the Netherlands and SWE for Sweden.

Logit regressions of the treatment effects reported in the main text and the supporting information are reported in Table E. For all model specifications, the dependent variable is an indicator variable, which is equal to one if the request triggered a response and zero otherwise. Models 2–5 include country controls. The difference in response rates for domestic sounding and Arabic sounding names is highly significant for the pooled data (Models 1 and 2), separated for the type of request (Model 3), separated for the rurality of the banks' location (Model 4), and controlling for both the type of request and the rurality of bank's location (Model 5). Marginal effects of discrimination rates are highly robust, varying between −22.7% and −23.7%, and highly significant for all model specifications. Differences in discrimination rates, for the type of request, and for the rurality of the banks' locations are statistically insignificant (as captured by the interaction terms *Ethnicity#Type* and *Ethnicity#Area*).

**Table E.** Logit regressions of the indicator variable *Response* on the ethnicity of the sender's name, the type of request, the rurality of the banks' location, and interaction effects.

| | (1)<br>Response | (2)<br>Response | (3)<br>Response | (4)<br>Response | (5)<br>Response |
|---|---|---|---|---|---|
| Ethnicity | $0.376^{***}$ | $0.361^{***}$ | $0.344^{***}$ | $0.388^{***}$ | $0.382^{***}$ |
| *[1 if foreign]* | (0.045) | (0.044) | (0.059) | (0.051) | (0.069) |
| | [−0.227] | [−0.227] | [−0.236] | [−0.237] | [−0.237] |
| Type (loan/investment) | | | $0.664^{*}$ | | $0.674^{*}$ |
| *[1 if investment]* | | | (0.111) | | (0.113) |
| | | | [−0.083] | | [−0.084] |
| Area (urban/rural) | | | | 0.669 | 0.685 |
| *[1 if rural]* | | | | (0.150) | (0.154) |
| | | | | [−0.140] | [−0.139] |
| Ethnicity#Type | | | 1.082 | | 1.029 |
| *[1 if foreign×investment]* | | | (0.265) | | (0.254) |
| Ethnicity#Area | | | | 0.527 | 0.501 |
| *[1 if foreign×rural]* | | | | (0.195) | (0.186) |
| Constant | $1.230^{*}$ | $0.671^{*}$ | 0.808 | 0.779 | 0.929 |
| | (0.100) | (0.136) | (0.176) | (0.164) | (0.209) |
| Country Controls | no | yes | yes | yes | yes |
| Observations | 1218 | 1218 | 1218 | 1218 | 1218 |
| Pseudo $R^2$ (McFadden) | 0.042 | 0.069 | 0.075 | 0.079 | 0.085 |
| Correct Predictions | 61.74% | 62.15% | 64.78% | 63.71% | 64.20% |
| $LR\ \chi^2(df)$ | 69.30 | 115.37 | 124.82 | 132.16 | 141.85 |

*Notes:* Logit regressions. Estimates are reported as odds ratios; marginal effects are reported in brackets. Standard errors are provided in parentheses. *, **, and *** refer to the 5%, 1%, and 0.1% significance level, respectively. # indicates an interaction term. Correct predictions report percentage of correctly predicted occurrences of the variable *Response* by the model. The model degrees of freedom, *df*, of likelihood ratio tests are 1, 7, 9, 9, and 11 for models 1–5, respectively.

## References

[1] Riach P, Rich J. Deceptive Field Experiments of Discrimination: Are they Ethical? Kyklos. 2004;57:457–470.
